# Histone H4 acetylation required for chromatin decompaction during DNA replication

**DOI:** 10.1038/srep12720

**Published:** 2015-07-30

**Authors:** Kun Ruan, Takaharu G. Yamamoto, Haruhiko Asakawa, Yuji Chikashige, Hiroshi Kimura, Hisao Masukata, Tokuko Haraguchi, Yasushi Hiraoka

**Affiliations:** 1 Graduate School of Frontier Biosciences, Osaka University, 1-3 Yamadaoka, Suita 565-0871, Japan; 2 Advanced ICT Research Institute Kobe, National Institute of Information and Communications Technology, 588-2 Iwaoka, Iwaoka-cho, Nishi-ku, Kobe 651-2492, Japan; 3 Department of Biological Sciences, Graduate School of Bioscience and Biotechnology, Tokyo Institute of Technology, 4259 Nagatsuda, Midori-ku, Yokohama 226-8501, Japan; 4 Department of Biological Sciences, Graduate School of Science, Osaka University, 1-1 Machikaneyama-cho, Toyonaka 560-0043, Japan

## Abstract

Faithful DNA replication is a prerequisite for cell proliferation. Several cytological studies have shown that chromosome structures alter in the S-phase of the cell cycle. However, the molecular mechanisms behind the alteration of chromosome structures associated with DNA replication have not been elucidated. Here, we investigated chromatin structures and acetylation of specific histone residues during DNA replication using the meiotic nucleus of the fission yeast *Schizosaccharomyces pombe*. The *S. pombe* meiotic nucleus provides a unique opportunity for measuring the levels of compaction of chromatin along the chromosome in a defined orientation. By direct measurement of chromatin compaction in living cells, we demonstrated that decompaction of chromatin occurs during meiotic DNA replication. This chromatin decompaction was suppressed by depletion of histone acetyltransferase Mst1 or by arginine substitution of specific lysine residues (K8 and K12) of histone H4. These results suggest that acetylation of histone H4 residues K8 and K12 plays a critical role in loosening chromatin structures during DNA replication.

The eukaryotic genome is organized into chromatin. Faithful meiotic DNA replication is a prerequisite for transmitting the genome from parent to offspring. Several cytological studies using a variety of experimental methods have supported a model in which chromosome structures alter in the S-phase of the cell cycle^1^,^2^,^3^,^4^. Studies using a premature chromosome condensation technique provided the first visual evidence for decondensation of chromatin fibers in S-phase^1^,^2^. Analysis of replication fork dynamics in living cells also suggests that the sites of DNA synthesis are sites of decondensed chromatin^4^. Posttranslational modification of histones, such as acetylation of histones H3 and H4, has been reported to regulate DNA replication^5^,^6^,^7^ probably by affecting chromatin structures^8^. However, the molecular mechanisms underlying the DNA replication-associated alteration of chromatin structures remain unknown.

The meiotic nucleus in the fission yeast *Schizosaccharomyces pombe* provides a useful experimental system in which to study chromatin structures in a defined orientation of chromosomes (Fig. 1A). In this organism, the nucleus elongates and moves back and forth between the cell ends upon entering meiosis (Supplementary Fig. S1A), and telomeres remain clustered at the leading edge of the moving nucleus; this elongated nucleus is generally called a “horsetail” nucleus due to its shape^9^,^10^. This unique arrangement of chromosomes in the horsetail nucleus has allowed direct measurements of chromatin compaction in living cells^11^. In our previous study, we measured chromatin compaction to demonstrate the function of meiotic cohesin Rec8 in chromosome compaction during meiotic prophase^11^.

Taking an advantage of this characteristic nuclear morphology in *S. pombe* meiosis, we previously isolated a *csn1* mutant in a screening of sporulation-deficient mutants that exhibits aberrant nuclear morphology^12^. The *csn1*^+^ gene encodes the subunit 1 of COP9 signalosome complex^13^, which is required for activity of the Pcu4-Ddb1^Cdt2^ E3 ubiquitin ligase complex^14^,^15^; COP9 signalosome complex and Pcu4-Ddb1^Cdt2^ ubiquitin ligase are required for degradation of Spd1, an inhibitor of ribonucleotide reductase (RNR). Because RNR activity is necessary to convert ribonucleotides to deoxyribonucleotides (dNTPs), deficiency of Csn1 causes stalled DNA replication due to inefficiency of dNTP synthesis^14^,^15^. In the current study, we measured chromatin compaction in the *csn1* mutant, and identified an association between histone H4 modification and chromatin compaction during DNA replication.

## Results

### Decompaction of chromatin occurs in mutants with stalled DNA replication

**Figure 1:**
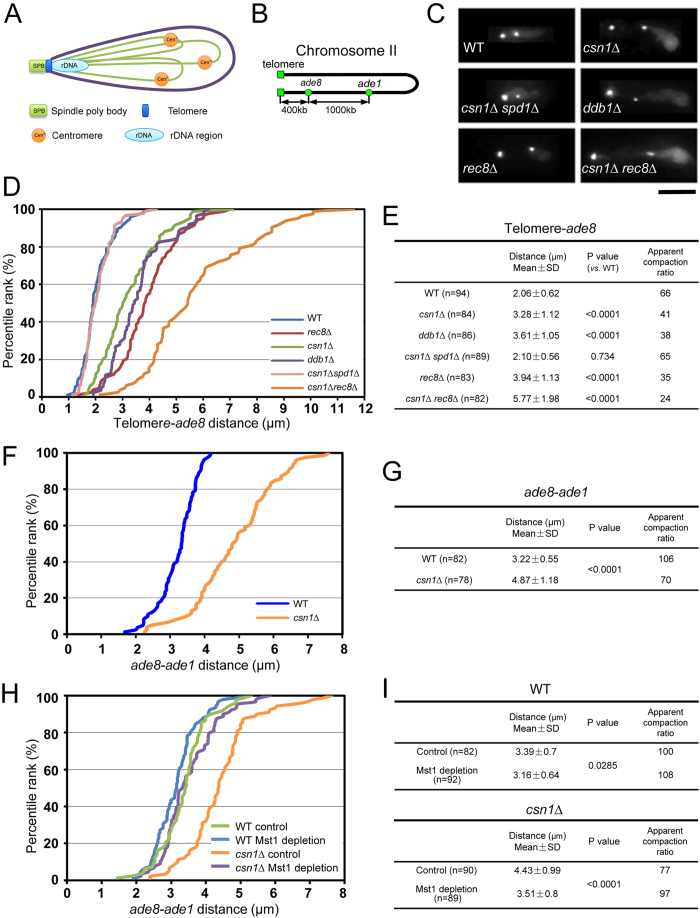
Measurements of chromatin compaction levels in living cells. (**A**) Schematic diagram of the meiotic nucleus in *S. pombe*. In meiotic prophase, the telomeres of all three chromosomes are clustered at the spindle-pole body^9^,^10^. Under the pulling force of the horsetail movement, the meiotic nucleus exhibits an elongated morphology. (**B**) Schematic diagram of the *lacO* inserts (*ade8* and *ade1* loci) on chromosome II. (**C**) Live-cell imaging of chromosomal loci in the horsetail nucleus: wild type (WT), *csn1*Δ, *csn1*Δ*spd1*Δ, *ddb1*Δ, *rec8*Δ, and *csn1*Δ*rec8*Δ. Telomeres were marked with Taz1-GFP; the *ade8* locus was visualized using *lacO*/LacI-GFP. Scale bar indicates 5 μm. (**D**,**F**,**H**) A percentile rank plot of distances between the telomere and *ade8* locus (**D**) or between the *ade8* and *ade1* loci (**F, H**) in the horsetail nucleus. (**E**,**G**,**I**) Statistical analysis of the distances measured in (**D**), (**F**), and (**H**). Means and standard deviations (SD) of the distances are shown. P values were calculated by Student’s t-test. A chromatin compaction ratio is defined as the length of DNA divided by the longitudinal length of chromatin. The 400-kb telomere-*ade8* chromatin region contains 136 μm of DNA; the 1,000-kb *ade8*-*ade1* chromatin region contains 340 μm of DNA

A *csn1*-deletion strain (*csn1∆*) exhibited a characteristic phenotype during nuclear movements^12^ similar to that reported in *rec8∆* strain^11^: the tip of the horsetail nucleus moved back and forth within the cell while the bulk of the nucleus did not follow the movements (Supplementary Fig. S1B), implying that the chromatin was stretched under the pulling forces of horsetail movement. To quantify the degree of chromatin compaction, we measured the distances between two loci along the chromosome (the telomere, the *ade8* locus or the *ade1* locus) in the horsetail nucleus in living cells, using a telomere protein, Taz1, tagged with green fluorescent protein (Taz1-GFP), and the *ade8* or *ade1* locus tagged with the *lacO*/LacI-GFP system on chromosome II (Fig. 1B). The average distance between the telomere and *ade8* locus in *csn1∆* cells was 1.6-fold greater than in wild-type cells (Fig. 1C–E). The average distance between the *ade8* locus and the *ade1* locus in *csn1∆* cells was also 1.5-fold greater than in wild-type cells (Fig. 1F,G), suggesting that chromatin is less compact in *csn1∆* than in wild-type cells. The degree of chromatin compaction can be estimated by calculating a chromatin compaction ratio, which is defined as the length of DNA contained between two loci divided by the measured longitudinal length of the respective region of chromatin.

Because Csn1 is required for activity of the Pcu4-Ddb1^Cdt2^ ubiquitin ligase complex^14^,^15^, cells deleted for the *ddb1*^+^ gene (*ddb1∆*) are expected to exhibit phenotypes similar to *csn1∆* cells. Thus, we examined nuclear morphology and measured the telomere-*ade8* distance in *ddb1∆* cells. As expected, the same phenotype of chromatin decompaction in the telomere-*ade8* region was observed in *ddb1∆* cells (Fig. 1C–E). Next we examined whether disruption of the *spd1*^+^ gene (*spd1∆*) could suppress formation of the abnormal horsetail nucleus, because the *spd1∆* mutation is known to suppress meiotic arrest in *csn1∆* and *ddb1∆* cells^15^. The Spd1 protein is an inhibitor of RNR and degradation of Spd1 through ubiquitin pathway involving Csn1 and Ddb1 is required for the effective synthesis of dNTPs upon reaching the S phase. Results showed that deletion of the *spd1*^+^ gene suppressed chromatin decompaction in *csn1∆* cells, as indicated by measurements of the telomere-*ade8* distance (Fig. 1C–E). Taken together, these results suggest that decompaction of chromatin observed in the *csn1∆* or *ddb1∆* mutant is due to stalled DNA replication caused by insufficiency of dNTPs.

Since it is known that the loss of Rec8 causes chromatin decompaction in meiotic prophase^11^, we also measured the telomere-*ade8* distance in *rec8∆* and *csn1∆ rec8∆* cells (Fig. 1C–E). The telomere-*ade8* distance in *rec8∆* was similar to that in *csn1∆* and *ddb1∆* cells (Fig. 1D,E). On the other hand, the distance in the *csn1∆ rec8∆* double mutant was greater than in each single mutant, indicating that the chromosomes were more extended in *rec8∆* under stalled DNA replication. These results suggest that stalled DNA replication has an additive effect, independent of cohesin function, on chromatin decompaction.

### Chromatin decompaction is suppressed by depletion of histone acetyltransferase Mst1

We considered that the observed chromatin decompaction might involve specific chromatin modifications. Since some histone acetylations are coupled with DNA replication^16^,^17^,^18^, we searched for a histone acetyltransferase (HAT) that affects chromatin decompaction in the horsetail nucleus in *csn1*∆ cells. For this purpose, we constructed strains in which each of five non-essential HAT genes (*hat1*^+^, *elp3*^+^, *gcn5*^+^, *rtt109*^+^, and *mst2*^+^) was disrupted, or an essential HAT, Mst1, was conditionally depleted in the *csn1∆* background, and examined whether stretched morphology of the horsetail nucleus was suppressed in these strains. Whereas disruption of the non-essential HAT genes did not affect stretching of the horsetail nucleus (Supplementary Fig. S2A), depletion of Mst1 suppressed it in *csn1∆* cells. Mst1, tagged with GFP-fused degron protein IAA17-GFP, was depleted using an improved-AID (*i*-AID) system^19^ (see Methods). Depletion of Mst1-IAA17-GFP was confirmed by fluorescence microscopy. To quantify the chromatin compaction levels, we measured the *ade*8-*ade1* distance in wild-type and *csn1∆* cells under conditions of Mst1 depletion. The *ade8-ade1* distance was distinctly shortened in *csn1∆* cells as a result of Mst1 depletion, while it was slightly shortened in wild-type cells (Fig. 1H; summarized in Fig. 1I). This observation implies that histone acetylation catalyzed by Mst1 is responsible for chromatin decompaction in *csn1∆* cells.

### Acetylation of histone H4 is necessary for chromatin decompaction during DNA replication

**Figure 2:**
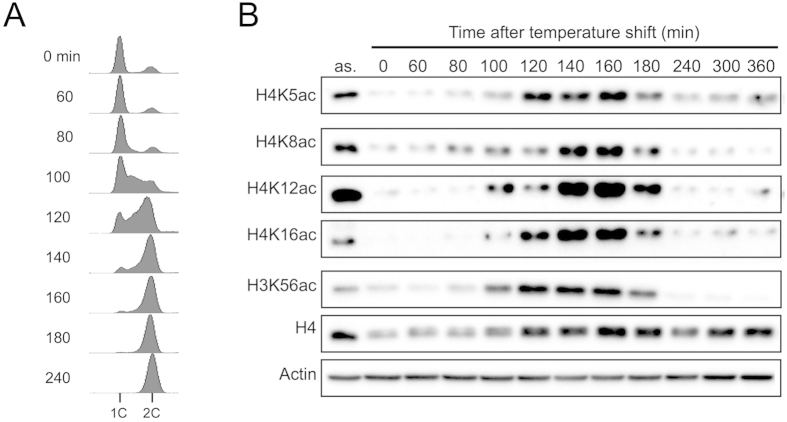
Acetylation of histone H4 during meiotic S phase. Haploid *h*^–^ *pat1-114* cells were cultured in nitrogen-free medium (EMM2-N) overnight at 26 °C. After 20 h, the culture was shifted to 34 °C to induce meiosis. (**A**) Flow cytometry of DNA contents during meiosis. Cells were sampled at the indicated time points for analysis. (**B**) Histone acetylation during meiosis. The cell extracts were prepared at the indicated time points and analyzed by immunoblotting. Actin was used as a loading control. Asynchronous cells (as) were obtained from preculture in YES medium.

As it is known that Mst1 catalyzes histone H4 acetylation in *S. pombe*^20^, we examined the acetylation of lysine residues (K5, K8, K12, and K16) of histone H4 during meiosis. Acetylation of histone H3-K56, which was reported as a replication-coupled histone modification^17^, was used as a positive control. The K5, K8, K12, and K16 residues of histone H4 were acetylated for 100–180 min after the induction of meiosis, which corresponds to the period of DNA replication (Fig. 2).

**Figure 3:**
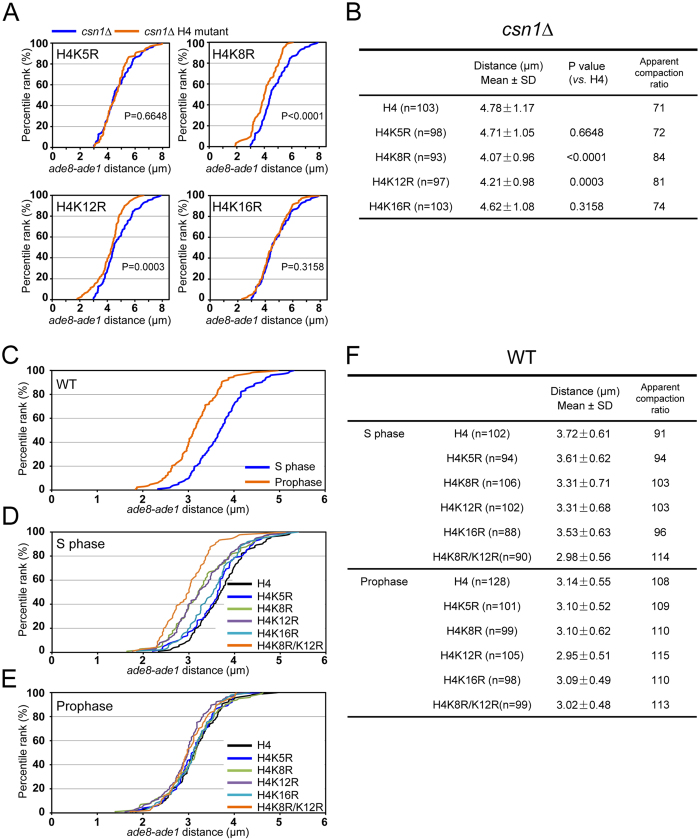
Chromatin decompaction and HU sensitivity in histone H4 mutants. Percentile rank plot of the *ade8*-*ade1* distance in *csn1∆* cells with the indicated mutation of histone H4 (orange). The plot for *csn1∆* expressing wild-type histone H4 (blue) was used as a control for all panels. (**B**) Statistical analysis of the distances measured in (**A**). (**C**) Percentile rank plot of the *ade8*-*ade1* distance in meiotic S phase and in meiotic prophase in the wild-type background. (**D**,**E**) Percentile rank plot of the *ade8*-*ade1* distance in meiotic S phase (**D**) and meiotic prophase (**E**) in cells expressing wild-type and mutant histone H4. (**F**) Statistical analysis of the distances measured in (**C**), (**D**), and (**E**). Means and standard deviations (SD) of the distances are shown. P values were calculated by Student’s t-test.

To identify the amino acid residues of histone H4 responsible for chromatin decompaction, we substituted each of four candidate lysine residues to arginine (K5R, K8R, K12R, and K16R). *S. pombe* has three copies of the histone H3-H4 gene cassette (*hht1*^+^-*hhf1*^+^*, hht2*^+^-*hhf2*^+^, and *hht3*^+^-*hhf3*^+^). To express a single copy of the mutated histone H4 gene, we mutated the *hhf2* gene in the background of *hht1*-*hhf1 hht3*-*hhf3* double deletion (see Methods). Expression of histone H4-K8R or -K12R partially suppressed chromatin decompaction in *csn1∆* cells, whereas expression of histone H4-K5R or -K16R did not (Supplementary Fig. S1C). These results were confirmed by measurements of the *ade8-ade1* distance: decompaction of chromatin in *csn1∆* cells was suppressed by histone H4-K8R or -K12R, but not by histone H4-K5R or -K16R (Fig. 3A,B). These results indicate that the decompaction of chromatin requires acetylation of K8 and K12 residues of histone H4. Although Mst1 also catalyzes Pht1 (histone H2A.Z) acetylation in *S. pombe*^21^, deletion of the *pht1*^+^ gene did not suppress stretching of the horsetail nucleus in *csn1∆* (Supplementary Fig. S2B), suggesting that histone H2A.Z is not involved in chromatin decompaction in these cells.

Since acetylation of histone H4 is involved in chromatin decompaction in *csn1∆* cells, chromatin might be loosened during meiotic DNA replication when histone H4 is acetylated. To test this possibility, we measured the *ade8-ade1* distance during S phase and prophase in living meiotic cells. Meiotic DNA replication occurs approximately at the beginning of the nuclear movements^12^,^22^,^23^. To distinguish S-phase cells from prophase cells, we used PCNA tagged with mCherry (mCherry-Pcn1) as a marker for DNA replication^12^,^24^. Cells containing the elongated horsetail nucleus with mCherry-Pcn1 foci represent S-phase cells and those without mCherry-Pcn1 foci represent meiotic prophase cells. In wild-type cells, the *ade8-ade1* distance was longer in S-phase cells than in meiotic prophase cells (Fig. 3C), indicating that chromatin decompaction indeed occurs during meiotic DNA replication. Cells expressing histone H4-K5R or -K16R showed distance profiles similar to wild-type cells in both S phase and prophase (Fig. 3D,E; summarized in Fig. 3F). In contrast, cells expressing histone H4-K8R or -K12R exhibited significant shortening of the *ade8-ade1* distance in S phase but not in prophase (Fig. 3D,E; summarized in Fig. 3F), suggesting that chromatin decompaction through histone H4 acetylation at K8 and K12 is specific to S phase. Furthermore, cells expressing histone H4 with mutations of both K8R and K12R (H4-K8R/K12R) exhibited more distinct shortening of the *ade8-ade1* distance in S phase in comparison to the single mutations (Fig. 3D). Thus, we conclude that acetylation of histone H4 at K8 and K12 is critical for loosening of chromatin structures during DNA replication.

### Acetylation of histone H4 at K8 and K12 residues is essential for vegetative growth

**Figure 4:**
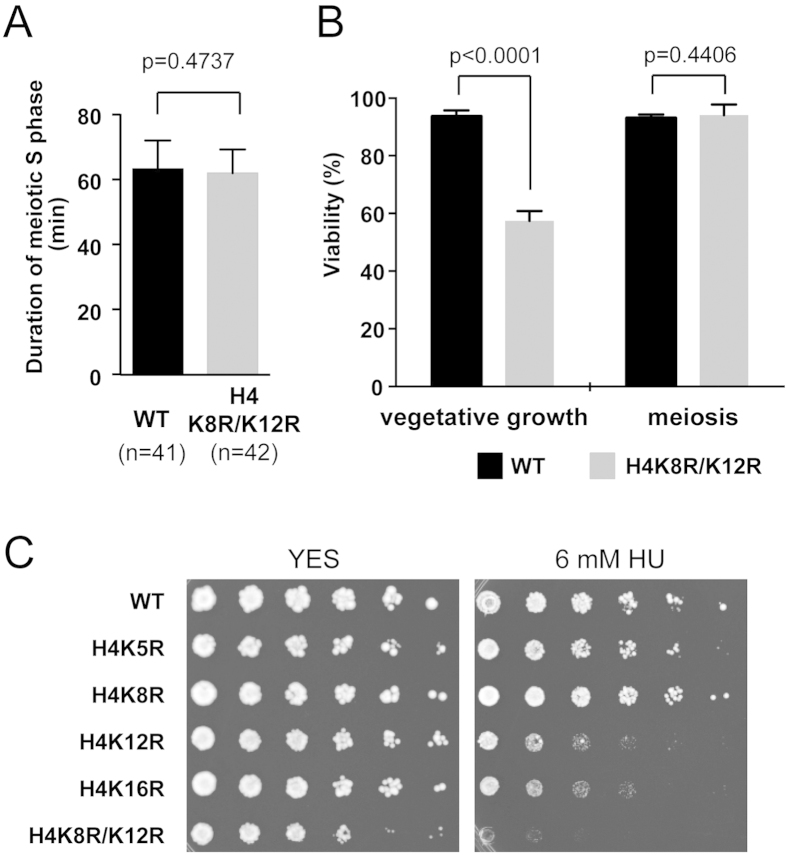
Acetylation of histone H4 at K8 and K12 residues is essential for vegetative growth. (**A**) Duration of meiotic S-phase in wild-type and histone H4-K8R/K12R cells. This experiment was performed as previous report^12^. Error bars represent the standard deviation. (**B**) Viability of vegetative cells and spores of wild-type and histone H4-K8R/K12R cells. Error bars represent the standard deviation. (**C**) HU sensitivity in cells expressing wild-type and mutant histone H4. Dilution series (1/5 dilution) of cell suspensions were spotted on complete medium (YES) with or without 6 mM HU and grown for three days at 30 °C.

To consider the possibility that the expression of mutant histone H4 affects the duration of the meiotic S phase, we measured the duration of meiotic DNA replication using GFP-Pcn1 in the histone H4-K8R/K12R mutant. The results showed that meiotic DNA replication was completed within the same duration in these mutant cells in which chromatin decompaction was not observed during meiotic DNA replication (Fig. 4A). In addition, there is no significant difference in spore viability between wild type and the histone H4-K8R/K12R mutant (Fig. 4B). Thus, the decompaction of chromatin is not absolutely necessary for meiotic DNA replication.

In contrast, vegetative cells expressing histone H4-K8R/K12R exhibited slow growth on a YES plate. Thus, we measured vegetative viability of these mutant cells. The viability of these mutant cells was only 57.5% compared with 93.6% in wild-type cells (Fig. 4B). It also should be noted that this mutant showed supersensitivity to hydroxyurea (HU), an inhibitor of RNR to decrease dNTP synthesis (Fig. 4C). These results suggest that acetylation of those histone H4 residues may necessary for vegetative growth in order to circumvent problems caused by stalled DNA replication. The difference in viability between vegetative cells and spores is an intriguing question, and remains to be elucidated.

## Discussion

In this study, our direct measurements of chromatin compaction in the meiotic horsetail nucleus of living *S. pombe* cells demonstrated that chromatin decompaction occurs during meiotic DNA replication. Our results also show that this decompaction is due to DNA replication-coupled acetylation of histone H4 by Mst1. DNA replication-coupled histone acetylation has also been reported in vegetative cells^16^,^17^,^18^; in addition, Mst1 interacts with MCM proteins^25^, suggesting that Mst1-mediated histone acetylation occurs during DNA replication in the mitotic cell cycle as well as in meiosis. Histone H4 acetylation may affect S-phase progression through regulation of gene expression. In fact, bromodomain-containing proteins bind to acetylated histones^26^,^27^ and regulate gene expression of some genes^28^,^29^. However, our measurements in living cells directly demonstrate that histone H4 acetylation alters chromatin compaction levels without affecting S-phase progression.

**Figure 5:**
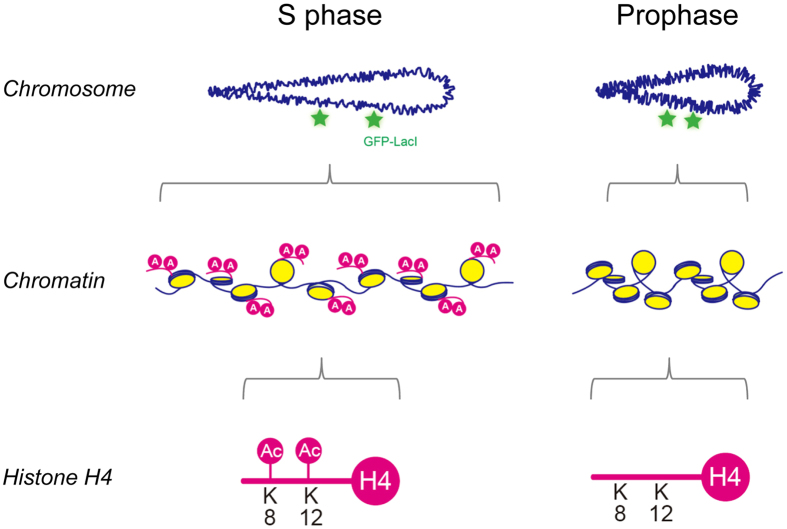
Histone acetylation associated with chromatin decompaction during DNA replication. Direct measurement of chromatin compaction demonstrates that acetylation at specific residues (K8 and K12) of histone H4 plays a critical role in chromatin decompaction during DNA replication.

We also identified the K8 and K12 residues of histone H4 as critical acetylation sites for chromatin decompaction during DNA replication (Fig. 5). In addition, the histone H4-K8R/K12R mutant is hypersensitive to HU, suggesting that acetylation of K8 and K12 of histone H4 play an important role in the response to DNA replication stress. However, a previous report showed that a histone H4 mutant bearing four substitutions (K5R, K8R, K12R, and K16R) had no effect on HU sensitivity^20^. This apparent discrepancy is probably due to the presence of two copies of the wild-type histone H4 gene in the reported mutant strains whereas our H4 mutant strains bear a mutated histone H4 gene as the sole histone H4 gene.

Interestingly, overproduction of Clr6, which catalyzes the deacetylation of histone H4, prevents phosphorylation of replication checkpoint Cds1 under DNA replication stress^30^. Therefore, defects in recovery of stalled DNA replication may be the result of impaired Cds1 function in the H4-K8R/K12R mutant. However, it has also been reported that high acetylation levels of histone H4 has a negative role in stabilization of replication forks in the absence of Cds1^20^. Thus, histone H4 acetylation plays multiple roles in the response to DNA replication stress.

Our results showed that the histone H4-K16R mutation did not affect chromatin decompaction during DNA replication. However, acetylation of H4-K16 is considered important for regulating the formation of higher-order chromatin structures^31^,^32^,^33^,^34^,^35^. The crystal structure of the nucleosome and *in vitro* chromatin assembly assays indicate residues 14–19 of histone H4 in the nucleosome interact with the acidic region on the surface of the adjacent nucleosome^31^,^32^,^33^. In addition, acetylation of histone H4-K16 prevents formation of 30-nm chromatin fibers^34^ while acetylation at histone H4-K5, K8, and K12 together only have a small additive effect on formation of the 30-nm fiber^35^. The difference between these *in vitro* assays and our *in vivo* assays may be because replication-coupled decompaction of chromatin occurs at a different order of chromatin organization instead of 30 nm fiber formation. In fact, the apparent compaction ratios of meiotic prophase chromatin are approximately 100, which is more compact than the ratio of 42 for the 30 nm fiber. Thus, the sites that are important for formation of 30 nm fibers may not be involved in the alteration of chromatin structures during DNA replication. Although further studies are required to address how acetylation of histone H4 regulates higher-order chromatin structures, our study clearly indicate a link between histone H4-K8/K12 acetylation and chromatin decompaction during DNA replication.

## Methods

### Cell culture and media

For routine culture, YES and EMM2 were used as complete medium and synthetic medium, respectively^36^. To induce meiosis, *h*^*90*^ mating-type cells were suspended in EMM2 without a nitrogen source (EMM2-N) and the suspension was spotted on ME agar plates. When *h*^+^ and *h*^–^ mating-type cells were used to induce meiosis, both strains were mixed in EMM2-N and the suspension was spotted on ME.

### Construction of strains and plasmids

The strains used in this study are listed in Table S1. Gene disruption and tagging with GFP, mRFP, or mCherry were performed as described^12^. The *i*-AID degron system was used for depletion of Mst1 protein^19^. To express skp1-AtTIR1-2NLS-9myc from the *lys1* gene locus, a 4.4-kb *Not*I fragment containing a *Padh15-skp1-AtTIR1-2NLS-9myc* fragment was obtained from pMK104^19^ and a *lys1-N* marker fragment was PCR-amplified from the pCST3 plasmid^23^. Both fragments were cloned into pBluescript II SK (-) (Stratagene), and the resulting plasmid pBlue-lys1-AtTIR1 was used for transformation. The transformants were selected on EMM2 plates lacking lysine, and used to tag Mst1 protein using IAA17-GFP. For IAA17-GFP tagging, the *IAA17* gene was PCR-amplified from the HM1905 genome^19^ and integrated into the *Sal*I site of pFA6-GFP (S65T)-kanMX6^37^ using an In-Fusion HD Cloning kit (Takara). The resulting plasmid was used to tag the endogenous *mst1* gene using a two-step PCR method^38^,^39^, and the constructed strain was used for Mst1-depletion experiments.

We constructed histone H4 mutant strains harboring only *hht2*-*hhf2* in the background of *hht1*-*hhf1 hht3*-*hhf3* double deletion as follows. To generate a mutated *hht2-hhf2* gene fragment, the entire *hht2-hhf2* locus (2.1 kb) was cloned by PCR from the wild-type genome (L968 strain). The fragment was inserted into a plasmid and a point mutation was introduced by site-directed mutagenesis using the PrimeSTAR Mutagenesis Basal Kit (Takara). After the mutation site was confirmed by sequencing, the resulting *hht2-hhf2* mutant gene fragment was used for transformation. To construct histone mutant strains, strains from which *hht2*^*+*^*-hhf2*^+^ had been deleted with a *ura4* marker in the background of *hht1*-*hhf1* deletion (RK1021) or *hht3*-*hhf3* deletion (HA1090-12D) were transformed with the mutant gene fragment. RK1021 and HA1090-12D were generated from YTP554^40^. Transformants were selected on YES agar containing 1 mg/mL 5-fluoroorotic acid (5-FOA). The mutations were confirmed by PCR and sequencing. Strains containing the mutated *hht2-hhf2* allele were crossed to obtain progeny harboring the *hht2-hhf2* mutant gene allele in the background of *hht1*-*hhf1* deletion and *hht3*-*hhf3* deletion.

### Fluorescence microscopy

A DeltaVision fluorescence microscopy system (Applied Precision), which is based on an Olympus wide-field IX71 fluorescence microscope equipped with an oil-immersion objective lens (Plan Apo 60×; NA = 1.4; Olympus) and a CoolSNAP HQ2 CCD camera (Photometrics), in a temperature-control room, was used for imaging of the fission yeast cells^41^. Cells were grown on YES agar at 30 °C or 26 °C for the *cdc10-129*^*ts*^ strain. To induce meiosis, the cells were transferred to an ME plate at 26 °C. After the formation of zygotes, the cells were suspended in EMM-N medium. Then, the cells were mounted on a 24 mm × 60 mm cover glass coated with 0.2% (w/v) lectin. Images were collected of 15 focal planes at 0.5 μm intervals. To measure the telomere-*ade8* or *ade8*-*ade1* distance, horsetail nuclei were observed in zygotes. The distance was measured only when the nucleus was moving straight in either direction (not making a turn). Time-lapse observation was performed as described^12^. Images were deconvolved with SoftWorx software (Applied Precision). The distance was measured using Priism software (University of California, San Francisco)^42^. Each trace was initiated at the center of the GFP focus.

### Induction of meiosis in Mst1-depleted conditions

For Mst1-depletion experiments, a *cdc10-129*^*ts*^ allele was used to arrest *csn1∆* cells at G1 phase before meiosis induction^43^,^44^ because *csn1∆* cells are difficult to arrest at the G1 phase by nitrogen starvation. *cdc10-129*^*ts*^ *csn1∆* cells were arrested for 4 h at the restrictive temperature of 36 °C to accumulate in G1. For the depletion of Mst1-IAA17-GFP protein, 0.5 mM auxin (1-naphthaleneacetic acid) (Nacalai Tesque) was added 2 h after the temperature shift to 36 °C. Then, the cells were mated on an auxin-containing EMM2-N agar plate at 26 °C. Depletion of Mst1-IAA17-GFP was confirmed by fluorescence microscopy.

### Viability assay of vegetative cells and spores

Log-phase cells cultured in YES were used for assay of vegetative viability. For spore viability assay, log-phase cells were washed twice with EMM2-N. Then the cells were spotted on ME plate and incubate at 26 °C for 2 days. Asci were digested by glucuronidase (Sigma) at 26 °C overnight. Cells or spores were separated manually using a micromanipulator on a tetrad microscope. For each sample, 120 cells or spores were inoculated on YES plates and cultured to form colonies at 30 °C for 7 days. Viability was determined from the number of colonies formed out of the number of cells or spores inoculated. Means and standard deviations were obtained from three measurements.

### Immunoblotting analysis

A total of 2.5 × 10^7^ asynchronously growing cells or 5 × 10^7^ G1-arrested cells were collected by centrifugation and the pellets were frozen at −80 °C. For immunoblotting, the cells were suspended in 800 μl ice-cold water containing 1 mM phenylmethylsulfonyl fluoride (PMSF) and disrupted with 150 μl of 2 M NaOH. Then, the cells were fixed with 150 μl of 55% trichloroacetate. After centrifugation, the pellets were dissolved in 200 μl of sample buffer (50 mM Tris-HCl [pH 6.8], 2% SDS, 12.5 mM EDTA, and 10% glycerol). Proteins in the extracts were separated by 15% SDS-PAGE and transferred to a PVDF membrane using a semidry system. The membranes were incubated for 1 h at room temperature in PBST buffer (10 mM Na_2_HPO_4_, 137 mM NaCl, 2.7 mM KCl, 1.76 mM KH_2_PO_4_, and 0.05% Tween-20) containing 5% skim milk. Then, the membranes were incubated with anti-actin antibody (Abcam), anti-H3K56 antibody (Millipore), anti-H4K5ac, anti-H4K8ac, anti-H4K12ac, anti-H4K16ac, or H4 antibody^45^ in PBST containing 1% skim milk at 4 °C overnight. After washing three times with PBST, the membranes were incubated with horseradish peroxidase-conjugated secondary antibodies (Millipore) in PBST containing 1% skim milk at room temperature for 1 h. The signals were visualized with ImmuneStar LD (Wako) and detected using Chemidoc MP imaging system (Bio-Rad).

### Flow cytometry

A total of 1 × 10^7^ cells were fixed with 70% ethanol and incubated with 50 μg/mL RNaseA in 500 μl of 50 mM sodium citrate (pH 7.5) for 4 h at 37°C, and then stained with 0.5 μg/mL propidium iodide. DNA content of 1.5 × 10^5^ cells was measured on a FACS Calibur instrument (BD Biosciences).

## Additional Information

**How to cite this article**: Ruan, K. *et al*. Histone H4 acetylation required for chromatin decompaction during DNA replication. *Sci. Rep*. **5**, 12720; doi: 10.1038/srep12720 (2015).

## Supplementary Material

Supplementary Information
