# Supplementary material for: Histone H4 acetylation required for chromatin decompaction during DNA replication
Source: Sci Rep. 2015 Jul 30;5:12720. doi: 10.1038/srep12720 (PMC4520004; doi:10.1038/srep12720)
Supplement: Supplementary Information [file srep12720-s1.pdf]

**Supplementary Information**

# Histone H4 acetylation required for chromatin decompaction during DNA replication

Kun Ruan[1], Takaharu G. Yamamoto[2], Haruhiko Asakawa[1], Yuji Chikashige[2], Hiroshi Kimura[3], Hisao Masukata[4], Tokuko Haraguchi[1, 2] and Yasushi Hiraoka[1, 2]*

[1]Graduate School of Frontier Biosciences, Osaka University, 1-3 Yamadaoka, Suita 565-0871, Japan; [2]Advanced ICT Research Institute Kobe, National Institute of Information and Communications Technology, 588-2 Iwaoka, Iwaoka-cho, Nishi-ku, Kobe 651-2492, Japan; [3]Department of Biological Sciences, Graduate School of Bioscience and Biotechnology, Tokyo Institute of Technology, 4259 Nagatsuda, Midori-ku, Yokohama 226-8501, Japan; [4]Department of Biological Sciences, Graduate School of Science, Osaka University, 1-1 Machikaneyama-cho, Toyonaka 560-0043, Japan

Running title: Histone H4 acetylation in *S. pombe*

Keywords: meiosis, DNA replication, chromatin, histone acetylation, fission yeast

*Corresponding author: Yasushi Hiraoka

Tel.: +81 6 6879 4621

Fax: +81 6 6879 4622

Email: hiraoka@fbs.osaka-u.ac.jp

## Table S1 Stain list

| Figures | Strain names | Genotypes | Sources |
|---|---|---|---|
| Figure 1 | AY278-11B | h$^{+}$ lys1-131 ura4-D18 his7$^{+}$::lacI-GFP | Ding *et al*., 2006 |
| | AY286-4A | h$^{-}$ ade6-216 leu1-32 ura4-D18 lys1$^{+}$::taz1-GFP taz1Δ::ura4$^{+}$ ade8[::kan$^{r}$-ura4$^{+}$-lacOp] his7$^{+}$::lacI-GFP | Ding *et al*., 2006 |
| | AY278-6A | h$^{+}$ lys1-131 ura4-D18 rec8Δ::ura4$^{+}$ his7$^{+}$::lacI-GFP | Ding *et al*., 2006 |
| | AY286-10C | h$^{-}$ leu1-32 ura4-D18 rec8Δ::ura4$^{+}$ lys1$^{+}$::taz1-GFP taz1Δ::ura4$^{+}$ ade8 [::kan$^{r}$-ura4$^{+}$-lacOp] his7$^{+}$::lacI-GFP | Ding *et al*., 2006 |
| | RK539 | h$^{-}$ ade6-216 leu1-32 ura4-D18 lys1$^{+}$::taz1-GFP taz1Δ::ura4$^{+}$ ade8[::kan$^{r}$-ura4$^{+}$-lacOp] his7$^{+}$::lacI-GFP ddb1Δ::hph | This work |
| | RK540 | h$^{+}$ lys1-131 ura4-D18 his7$^{+}$::lacI-GFP ddb1Δ::hph | This work |
| | RK901 | h$^{-}$ ade6-216 leu1-32 ura4-D18 lys1$^{+}$::taz1-GFP taz1Δ::ura4$^{+}$ ade8[::kan$^{r}$-ura4$^{+}$-lacOp] his7$^{+}$::lacI-GFP csn1Δ::hph | This work |
| | RK902 | h$^{+}$ lys1-131 ura4-D18 his7$^{+}$::lacI-GFP csn1Δ::hph | This work |
| | RK907 | h$^{-}$ ade6-216 leu1-32 ura4-D18 lys1$^{+}$::taz1-GFP taz1Δ::ura4$^{+}$ ade8[::kan$^{r}$-ura4$^{+}$-lacOp] his7$^{+}$::lacI-GFP csn1Δ::hph spd1Δ::nat$^{r}$ | This work |
| | RK908 | h$^{+}$ lys1-131 ura4-D18 his7$^{+}$::lacI-GFP csn1Δ::hph spd1Δ::nat$^{r}$ | This work |
| | RK1121 | h$^{-}$ ade6-216 his2 leu1-32 lys1-131 ura4-D18 ade1[::kan$^{r}$-ura4$^{+}$-lacOp] ade8[::kan$^{r}$-ura4$^{+}$-lacOp] his7$^{+}$::lacI-GFP aur1$^{r}$::htb1-mCherry | This work |
| | RK1130 | h$^{-}$ ade6-216 his2 leu1-32 lys1-131 ura4-D18 ade1[::kan$^{r}$-ura4$^{+}$-lacOp] ade8[::kan$^{r}$-ura4$^{+}$-lacOp] his7$^{+}$::lacI-GFP aur1$^{r}$::htb1-mCherry csn1Δ::nat$^{r}$ | This work |
| | RK1228 | h$^{-}$ leu1-32 ura4-D18 rec8Δ::ura4$^{+}$ lys1$^{+}$::taz1-GFP taz1::ura4$^{+}$ ade8[::kan$^{r}$-ura4$^{+}$-lacOp] his7$^{+}$::lacI-GFP csn1Δ::nat$^{r}$ | This work |
| | RK1229 | h$^{+}$ lys1-131 ura4-D18 rec8Δ::ura4$^{+}$ his7$^{+}$::lacI-GFP csn1Δ::nat$^{r}$ | This work |
| | RK1303 | h$^{90}$ ade6-210 leu1-32 ura4-D18 cdc10-129 lys1$^{+}$::Padh15-skp1-AtTIR1-2NLS aur1$^{r}$::htb1-mCherry mst1$^{+}$::IAA17-GFP-nat$^{r}$ ade1[::kan$^{r}$-ura4$^{+}$-lacOp] ade8[::kan$^{r}$-ura4$^{+}$-lacOp] his7$^{+}$::lacI-GFP | This work |
| | RK1331 | h$^{90}$ ade6-210 leu1-32 ura4-D18 cdc10-129 lys1$^{+}$::Padh15-skp1-AtTIR1-2NLS aur1$^{r}$::htb1-mCherry mst1$^{+}$::IAA17-GFP-ura4$^{+}$ ade1[::kan$^{r}$-ura4$^{+}$-lacOp] ade8[::kan$^{r}$-ura4$^{+}$-lacOp] his7$^{+}$::lacI-GFP csn1Δ::hph | This work |
| Figure 2 | RK1085 | h$^{-}$ pat1-114 ura4 lys1$^{+}$::Padh15-skp1-AtTIR1-2NLS | This work |
| Figure 3 | RK1481 | h$^{90}$ ade6-216 leu1-32 lys1-131 ade1[::kan$^{r}$-ura4$^{+}$-lacOp] ade8[::kan$^{r}$-ura4$^{+}$-lacOp] his7$^{+}$::lacI-GFP hht1-hhf1Δ::kan$^{r}$ csn1Δ::hph hht3-hhf3Δ::nat$^{r}$ hht2-hhf2K5R | This work |

| | | | |
|---|---|---|---|
| | RK1482 | $h^{90}$ ade6-216 leu1-32 lys1-131 ade1[::kan$^{r}$-ura4$^{+}$-lacOp] ade8[::kan$^{r}$-ura4$^{+}$-lacOp] his7$^{+}$::lacI-GFP hht1-hhf1Δ:: kan$^{r}$ csn1Δ::natr hht3-hhf3Δ::nat$^{r}$ hht2-hhf2K12R | This work |
| | RK1483 | $h^{90}$ ade6-216 leu1-32 lys1-131 ura4-D18 ade1[::kan$^{r}$-ura4$^{+}$-lacOp] ade8[::kan$^{r}$-ura4$^{+}$-lacOp] his7$^{+}$::lacI-GFP hht1-hhf1Δ:: kan$^{r}$ csn1Δ::hph hht3-hhf3Δ::nat$^{r}$ hht2-hhf2K8R | This work |
| | RK1484 | $h^{90}$ leu1-32 lys1-131 ade1[::kan$^{r}$-ura4$^{+}$-lacOp] ade8[::kan$^{r}$-ura4$^{+}$-lacOp] his7$^{+}$::lacI-GFP hht1-hhf1Δ:: kan$^{r}$ csn1Δ::nat$^{r}$ hht3-hhf3Δ::nat$^{r}$ hht2-hhf2K16R | This work |
| | RK1532 | $h^{90}$ ade6-216 leu1-32 lys1-131 ade1[::kan$^{r}$-ura4$^{+}$-lacOp] ade8[::kan$^{r}$-ura4$^{+}$-lacOp] his7$^{+}$::lacI-GFP hht1-hhf1Δ:: kan$^{r}$ csn1Δ::nat$^{r}$ hht3-hhf3Δ::nat$^{r}$ | This work |
| | RK1590 | $h^{90}$ ade6-216 leu1-32 lys1-131 ade1[::kan$^{r}$-ura4$^{+}$-lacOp] ade8[::kan$^{r}$-ura4$^{+}$-lacOp] his7$^{+}$::lacI-GFP hht1-hhf1Δ:: kan$^{r}$ hht3-hhf3Δ::hph aur1$^{r}$ : Pnda3-mCherry-pcn1$^{+}$ | This work |
| | RK1591 | $h^{90}$ leu1-32 lys1-131 ura4-D18 ade1[::kan$^{r}$-ura4$^{+}$-lacOp] ade8[::kan$^{r}$-ura4$^{+}$-lacOp] his7$^{+}$::lacI-GFP hht2-hhf2K8R/K12R hht1-hhf1Δ::ble$^{r}$ hht3-hhf3Δ::hph aur1$^{r}$ :Pnda3-mCherry-pcn1$^{+}$ | This work |
| | RK1597 | $h^{90}$ ade6-216 leu1-32 lys1-131 ade1[::kan$^{r}$-ura4$^{+}$-lacOp] ade8[::kan$^{r}$-ura4$^{+}$-lacOp] his7$^{+}$::lacI-GFP hht1-hhf1Δ::ble$^{r}$ hht3-hhf3Δ::hph hht2-hhf2K5R aur1$^{r}$::Pnda3-mCherry-pcn1$^{+}$ | This work |
| | RK1598 | $h^{90}$ leu1-32 lys1-131 ade1[::kan$^{r}$-ura4$^{+}$-lacOp] ade8[::kan$^{r}$-ura4$^{+}$-lacOp] his7$^{+}$::lacI-GFP hht1-hhf1Δ:: kan$^{r}$ hht3-hhf3Δ::hph hht2-hhf2K12R aur1$^{r}$::Pnda3-mCherry-pcn1$^{+}$ | This work |
| | RK1599 | $h^{90}$ ade6-216 leu1-32 lys1-131 ura4-D18 ade1[::kan$^{r}$-ura4$^{+}$-lacOp] ade8[::kan$^{r}$-ura4$^{+}$-lacOp] his7$^{+}$::lacI-GFP hht1-hhf1Δ::ble$^{r}$ hht3-hhf3Δ::hph hht2-hhf2K8R aur1$^{r}$::Pnda3-mCherry-pcn1$^{+}$ | This work |
| | RK1600 | $h^{90}$ ade6-216 leu1-32 lys1-131 ade1[::kan$^{r}$-ura4$^{+}$-lacOp] ade8[::kan$^{r}$-ura4$^{+}$-lacOp] his7$^{+}$::lacI-GFP hht1-hhf1Δ:: kan$^{r}$ hht3-hhf3Δ::hph hht2-hhf2K16R aur1$^{r}$::Pnda3-mCherry-pcn1$^{+}$ | This work |
| **Figure 4** | RK1201 | $h^{90}$ ura4-D18 leu1-32 lys1-131 hht1-hhf1Δ::kan$^{r}$ hht3-hhf3Δ::nat$^{r}$ aur1$^{r}$::htb1-mCherry | This work |
| | RK1202 | $h^{90}$ ura4-D18 leu1-32 lys1-131 hht1-hhf1Δ::kan$^{r}$ hht2-hhf2K5R hht3-hhf3Δ::nat$^{r}$ aur1$^{r}$::htb1-mCherry | This work |
| | RK1206 | $h^{90}$ ura4-D18 leu1-32 lys1-131 hht1-hhf1Δ::kan$^{r}$ hht2-hhf2K8R hht3-hhf3Δ::nat$^{r}$ aur1$^{r}$::htb1-mCherry | This work |
| | RK1281 | $h^{90}$ ura4-D18 leu1-32 lys1$^{+}$::Ppcn1-GFP-pcn1$^{+}$ hht1-hhf1Δ::kan$^{r}$ hht3-hhf3Δ::nat$^{r}$ aur1$^{r}$::htb1-mCherry | This work |
| | RK1320 | $h^{90}$ ura4-D18 leu1-32 lys1-131 hht1-hhf1Δ::kan$^{r}$ hht2-hhf2K8/K12R hht3-hhf3Δ::nat$^{r}$ | This work |
| | RK1333 | $h^{90}$ ura4-D18 leu1-32 lys1-131 hht1-hhf1Δ::kan$^{r}$ hht2-hhf2K12R hht3-hhf3Δ::nat$^{r}$ aur1$^{r}$::htb1-mCherry | This work |
| | RK1334 | $h^{90}$ ura4-D18 leu1-32 lys1-131 hht1-hhf1Δ:kan$^{r}$ | This work |

| | | | |
|---|---|---|---|
| | | *hht2-hhf2K16R hht3-hhf3Δ::nat$^r$ aur1$^r$::htb1-mCherry* | |
| | RK1335 | *h$^{90}$ ura4-D18 leu1-32 lys1-131 hht1-hhf1Δ::kan$^r$ hht2-hhf2K8/K12R hht3-hhf3Δ::nat$^r$ aur1$^r$::htb1-mCherry* | This work |
| | RK1352 | *h$^{90}$ ura4-D18 leu1-32 lys1$^+$::Ppcn1-GFP-pcn1$^+$ hht1-hhf1Δ::kan$^r$ hht2-hhf2K8/K12R hht3-hhf3Δ::nat$^r$ aur1$^r$::htb1-mCherry* | This work |
| **Figure S1** | RK1577 | *h$^{90}$ ura4-D18 leu1-32 lys1$^+$::htb1-GFP hht1-hhf1Δ::kan$^r$ Dhht3-hhf3Δ::nat$^r$* | This work |
| | RK1243 | *h$^{90}$ ura4-D18 leu1-32 lys1$^+$::htb1-GFP hht1-hhf1Δ::kan$^r$ hht3-hhf3Δ::nat$^r$ csn1Δ::hph* | This work |
| | RK1245 | *h$^{90}$ ura4-D18 leu1-32 lys1$^+$::htb1-GFP hht1-hhf1Δ::kan$^r$ hht2-hhf2K8R hht3-hhf3Δ::nat$^r$ csn1Δ::hph* | This work |
| | RK1258 | *h$^{90}$ ade6-216 ura4-D18 leu1-32 lys1$^+$::htb1-GFP mes1::LEU2 hht1-hhf1Δ::kan$^r$ hht2-hhf2K5R csn1Δ::hph hht3-hhf3Δ::nat$^r$* | This work |
| | RK1260 | *h$^{90}$ ura4-D18 leu1-32 lys1$^+$::htb1-GFP hht1-hhf1Δ::kan$^r$ hht2-hhf2K12R csn1Δ::hph hht3-hhf3Δ::nat$^r$* | This work |
| | RK1261 | *h$^{90}$ ura4-D18 leu1-32 lys1$^+$::htb1-GFP hht1-hhf1Δ::kan$^r$ hht2-hhf2K16R csn1Δ::hph hht3-hhf3Δ::nat$^r$* | This work |
| **Figure S2** | RK478 | *h$^{90}$ ade6-210 leu1-32 lys1-131 ura4-D18 hht1-mRFP-hph* | This work |
| | RK747 | *h$^{90}$ ade6-210 leu1-32 lys1-131 ura4-D18 hht1-mRFP-hph rtt109Δ::kan$^r$ csn1Δ::ura4$^+$* | This work |
| | RK750 | *h$^{90}$ ade6-210 leu1-32 lys1-131 ura4-D18 hht1-mRFP-hph mst2Δ::kan$^r$ csn1Δ::nat$^r$* | This work |
| | RK753 | *h$^{90}$ ade6-210 leu1-32 lys1-131 ura4-D18 hht1-mRFP-hph elp3Δ::kan$^r$ csn1Δ::nat$^r$* | This work |
| | RK933 | *h$^{90}$ hht1-mRFP-kanr pht1Δ::nat$^r$ csn1Δ::hph* | This work |
| | RK1309 | *h$^{90}$ ade6-210 leu1-32 ura4-D18 lys1$^+$::htb1-mCherry hat1Δ::hph csn1Δ::nat$^r$* | This work |
| | RK1310 | *h$^{90}$ ade6-210 leu1-32 ura4-D18 lys1$^+$::htb1-mCherry gcn5Δ::hph csn1Δ::nat$^r$* | This work |

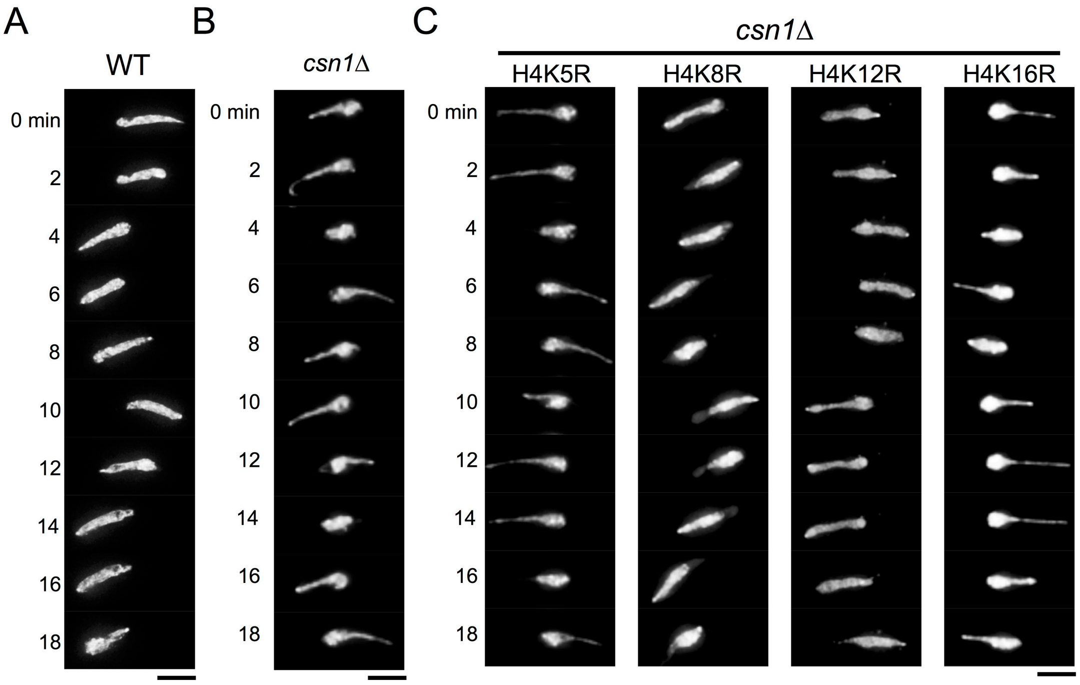


**Figure S1. Morphology of the horsetail nucleus in *csn1Δ* cells expressing mutant histone H4**

Time-lapse imaging of the horsetail nucleus. Chromatin was marked with Htb1 (histone H2B)-GFP. Numbers on the left indicate time in minutes. Scale bars indicate 5 $\mu$m. (**A**) Wild type, (**B**) *csn1Δ* expressing wild-type histone H4, and (**C**) *csn1Δ* expressing histone H4 mutants as indicated.

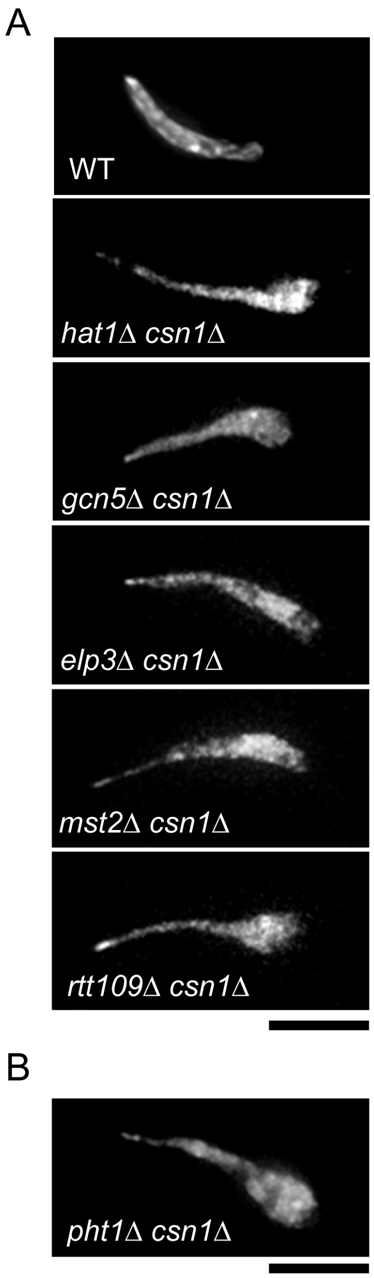


**Figure S2. The stretched horsetail nucleus in HAT and H2A.Z mutants**

(**A**) Morphology of the horsetail nucleus in the *csn1Δ* HAT double mutants. (**B**) Morphology of the horsetail nucleus in the *csn1Δ pht1Δ* double mutant. Chromatin was marked with Hht1 (histone H3)-mRFP. Scale bar indicates 5 $\mu$m.
